# Ozonized Hydrogels vs. 1% Chlorhexidine Gel for the Clinical and Domiciliary Management of Peri-Implant Mucositis: A Randomized Clinical Trial

**DOI:** 10.3390/jcm12041464

**Published:** 2023-02-12

**Authors:** Andrea Butera, Maurizio Pascadopoli, Simone Gallo, Carlos Pérez-Albacete Martínez, José Eduardo Maté Sánchez de Val, Luca Parisi, Alice Gariboldi, Andrea Scribante

**Affiliations:** 1 Unit of Dental Hygiene, Section of Dentistry, Department of Clinical, Surgical, Diagnostic and Pediatric Sciences, University of Pavia, 27100 Pavia, Italy; 2 Health Sciences PhD Programme, Universidad Catòlica de Murcia UCAM, Campus de Ios Jerònimos N. 135, Guadalupe, 30107 Murcia, Spain; 3 Unit of Orthodontics and Pediatric Dentistry, Section of Dentistry, Department of Clinical, Surgical, Diagnostic and Pediatric Sciences, University of Pavia, 27100 Pavia, Italy; 4 Tissue Regeneration and Repair Group, Biomaterials and Tissue Engineering, Faculty of Health Sciences, UCAM—Universidad Catòlica San Antonio de Murcia, Guadalupe, 30107 Murcia, Spain; 5 Department of Biomedical, Surgical and Dental Sciences, University of Milan, 20122 Milan, Italy

**Keywords:** ozone, ozonated oils, peri-implant mucositis, chlorhexidine, scaling and root planing, implants, periodontal parameters, randomized clinical trial

## Abstract

Peri-implant mucositis consists of a reversible inflammation of peri-implant tissues characterized by bleeding on gentle probing in the absence of bone loss. Ozone therapy is being extensively studied for its efficacy in treating different dental conditions. To date, few studies have evaluated ozone as an adjunct to the oral hygiene measures of peri-implant mucositis patients. The aim of the present study is to assess the efficacy of an ozonized gel (Trial group) compared to chlorhexidine (Control group) after a domiciliary protocol of oral hygiene in a 6-month study. According to a split-mouth study design, patients were divided into Group 1 for the application of chlorhexidine gel in peri-implant mucositis sites of quadrants Q1 and Q3, whereas in quadrants Q2 and Q4, the ozonized gel was in-office administered. For Group 2, the quadrants were inverted. At baseline (T0), and after 1 (T1), 2 (T2), and 3 (T3) months, Probing Depth (PD), Plaque Index (PI), SI Suppuration Index (SI), Bleeding Score (BS) and Marginal Mucosa Condition (MMC) were measured. A statistically significant decrease was found for all the variables assessed in each group (*p* < 0.05), whereas significant intergroup differences were found only for PI, BoP, and BS. Accordingly, both agents tested in this study showed an efficacy in treating peri-implant mucositis. The ozonized gel deserves particular attention, considering the better outcome than chlorhexidine on specific clinical periodontal parameters, as well as its lesser shortcomings.

## 1. Introduction

**Figure 1:**
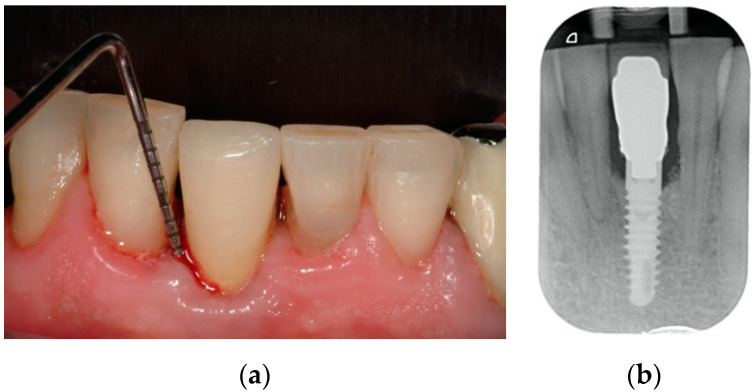
Peri-implant mucositis site of a lower right central incisor: (**a**) sign of bleeding on probing, and (**b**) radiographical evaluation showing no bone loss around implant collar.

Peri-implant mucositis is a reversible plaque-related peri-implant disease characterized by bleeding on gentle probing in the absence of the loss of supporting peri-implant bone (Figure 1). Erythema, swelling, and suppuration signs can also be present [1]. With the loss of periodontal tissues, progression toward peri-implantitis can occur, leading to implant failure [2].

According to a recent systematic review with meta-analysis, the estimated prevalence of peri-implant mucositis is 29.48% at the implant level and 46.83% at the patient level [3]. Current evidence indicates that regular professional oral hygiene and domiciliary maneuvers are the gold standard for patients with implant-supported prosthesis [4]. In addition to standard protocols, adjunctive treatments with lasers, probiotics, and local and systemic antibiotics have also been investigated, but they seem to be effective only for peri-implantitis [5]. However, there is low evidence due to the scarcity of research on the topic.

Well-known antibacterial and healing properties led to the commercialization of gaseous ozone and ozonated water devices, together with ozonated oil, in various segments of dental practice [6]. Even though it seems that no effects can be obtained by using ozone as an adjunct to non-surgical treatment [7], its use on peri-implant tissue was not yet explored, with little literature on clinical studies published in this field [8].

Therefore, given the scarcity of studies evaluating an ozonized oil-based gel as an adjunct to the oral hygiene measures of peri-implant mucositis patients, the purpose of the current report was to assess the efficacy of an ozonized hydrogel (Trial group) compared to chlorhexidine (Control group) after a domiciliary protocol of oral hygiene in a 6-month study.

The statistically null hypothesis of the research was that no significant differences in the clinical periodontal indexes evaluated would be found between the two groups.

## 2. Materials and Methods

### 2.1. Trial Design

This was a single-center, split-mouth randomized controlled trial with a 1:1 allocation ratio, approved by the Unit Internal Review Board (registration number: 2022-0112) and registered on Clinicaltrials.gov (NCT number: NCT05256914).

### 2.2. Participants 

The study was conducted at the Unit of Dental Hygiene, Section of Dentistry, Department of Clinical, Surgical, Diagnostic and Pediatric Sciences of the University of Pavia (Pavia, Italy), starting in February 2022 and ending in October 2022. Patients were asked to sign an informed consent document before participating. Both the interventions and the outcomes assessments were conducted at the same unit.

The inclusion and exclusion criteria are listed in Table 1.

### 2.3. Interventions and Outcomes 

After signing the informed consent document (baseline, T0), patients were visited and the following indexes were collected by an instructed operator by means of a probe (UNC probe 15; Hu-Friedy, Chicago, IL, USA): PD, Probing Depth (distance between soft margin of the gum and base of the pocket) [9]; PI, Plaque Index (percentage of sites with plaque with respect to total dental sites) [10]; SI, Suppuration Index (presence or absence of suppuration in the peri-implant site); BS, Bleeding Score (presence of bleeding on probing on a scale of 0–3) [11]; and MMC, Marginal Mucosa Condition (presence of qualitative changes in the mucosa on a scale of 0–3) [9]. Then, a professional supragingival and subgingival oral hygiene appointment was conducted with a piezoelectric instrument (Multipiezo, Mectron S.p.a., Carasco, Italy), Gracey curettes (Hu-Friedy, Chicago, IL, USA), PEEK ultrasonic tip (Implant Cleaning Set S, Mectron S.p.a., Carasco, Italy), and a titanium curette for implant sites (Implant Curette TIS2CN, Arnold Deppeler SA, Rolle, Switzerland); this was followed by decontamination with glycine powder (Mectron S.p.a., Carasco, Italy).

According to the split-mouth study, patients were divided into two groups: Group 1 received the application of Curasept Periodontal Gel in peri-implant mucositis sites of quadrants Q1 and Q3, whereas for quadrants Q2 and Q4, Ozoral Pro was administered in-office. For Group 2, the quadrants were inverted. Table 2 shows the compositions of the two products used.

Following the professional oral hygiene appointment, each peri-implant mucositis site was rinsed, air-dried, and isolated by means of cotton rolls so that the assigned gel could be applied and left for at least 2 min. 

Patients were visited after 1 (T1), 3 (T2), and 6 (T3) months. All of the clinical procedures were repeated for each time frame, except for the professional oral hygiene appointment, which was repeated at T3 at the end of the visit. For the duration of the study, patients applied Curasept Periodontal Gel and Ozoral Gel to the same quadrants as the in-office administration once a day for the next 14 days (based on the recommended chlorhexidine protocol). Patients were given two different syringes with a blunt plastic needle of 5–6 mm in diameter for the domiciliary administration. 

### 2.4. Sample Size

The sample size calculation (alpha = 0.05; power = 95%) for two independent study groups and a continuous primary endpoint was calculated.

The following mathematical formula was used for the sample size calculation:

$$\mathrm{Sample}\;\mathrm{size}=\frac{Z_{\left(1-\frac{\alpha }{2}\right)}^{2}p\left(1-p\right)}{d^{2}}$$

where Z is the standard normal variate corresponding to 1.96 at 5% type 1 error, *p* is the expected proportion of the population expressed as a decimal and based on previous studies, and finally d is the confidence level determined by the researcher and expressed as a decimal, too.

The variable Probing Depth was chosen as the primary outcome. A mean of 3.35 was expected, and a difference between the means of 0.59, with a standard deviation of 1.10 [12]. Therefore, 90 peri-implant mucositis sites per group were required for the split-mouth study.

### 2.5. Randomization and Blinding

With a block randomization table, the data analyst generated a randomization sequence considering a permuted block of 90 peri-implant sites due to the split-mouth design. After the random assignment of the Trial treatment for one quadrant, the contralateral one was allocated to the Control treatment. Opaque envelopes were previously prepared, sealed, and numbered sequentially (SNOSE); afterward, an operator performed the procedures and the index collection after assigning the quadrants to the respective treatments. For the home oral hygiene procedures, the two gels were concealed. Patients and the data analyst were blinded for the allocation. For the domiciliary protocol the two gels had different colors to help the participants, and written instructions were left on the packaging to avoid mistakes due to the split-mouth design.

### 2.6. Statistical Methods

Data underwent statistical analysis with R Software (R version 3.1.3, R Development Core Team, R Foundation for Statistical Computing, Wien, Austria). Descriptive statistics (mean, standard deviation, minimum, median, and maximum) were calculated for each group and variable. The data normality of the distributions was calculated with the Kolmogorov–Smirnov test. The Friedman non-parametric test was then performed, followed by Dunn’s post hoc test.

Significance was predetermined as *p* < 0.05 for all the tests performed.

## 3. Results

### 3.1. Participant Flow and Baseline Data

**Figure 2:**
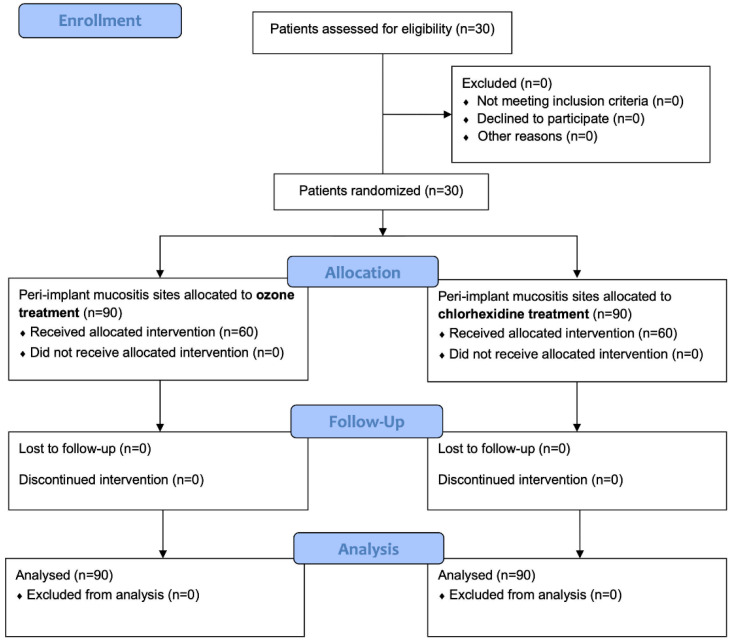
CONSORT flow chart of the study showing enrollment and allocation procedures.

Participants were enrolled until the required number of peri-implant mucositis sites was reached. In total, 30 patients were enrolled according to the inclusion criteria, and they agreed to participate and received the allocated interventions. No patient was excluded from the analysis. The flow chart of the study is shown in Figure 2. At the baseline (T0), the sample showed a mean age of 59.03 ± 8.31 years (14 females and 16 males). The characteristics of the study sample are shown in Table 3.

To show inter and intragroup differences, a letter-based comparison system was used. Letters were assigned to means, so the same letter/letters between groups denoted that no significant difference existed [13].

### 3.2. Probing Depth (PD)

PD scores are shown in Table 4. A significant decrease in PD values was found for both of the groups (*p* < 0.05). As regards intragroup differences, a gradual significant reduction in PD for both of the groups was found over all the time frames of the study (*p* < 0.05); no significant intergroup differences occurred (*p* > 0.05).

### 3.3. Plaque Index (PI)

PI scores are shown in Table 5. A significant decrease was found in both of the groups (*p* < 0.05). Intragroup comparisons showed significant differences in the interval T0–T1 in both of the groups (*p* < 0.05). A significant intergroup difference was found at T2, with lower scores for the Trial group (*p* < 0.05). 

### 3.4. Bleeding on Probing (BoP)

BoP scores are shown in Table 6. A significant reduction was found in both of the groups (*p* < 0.05). Significant intragroup differences were found in the Control group between T0–T1 and T1–T2 (*p* < 0.05), while they were found in the Trial group between all of the time frames (*p* < 0.05). Significant intergroup differences were found between the groups at T1, T2, and T3 (*p* < 0.05).

### 3.5. Suppuration Index (SI)

SI values are shown in Table 7. A significant decrease was found in both of the groups (*p* < 0.05). Intragroup comparisons highlighted a significant difference in the interval T2–T3 for the Control group and in the interval T1–T2 for the Trial group (*p* < 0.05). Intergroup comparisons showed no statistically significant differences between the groups for all of the time frames of the study (*p* > 0.05).

### 3.6. Bleeding Score (BS)

BS values are shown in Table 8. A gradual decrease was found in both of the groups (*p* < 0.05). Intragroup comparisons showed a significant decrease at the interval T1–T2 in the Control group (*p* < 0.05); in the Trial group, significant differences were found at T0–T1 and T1–T2 (*p* < 0.05). Intergroup comparisons highlighted a significant difference between the groups at T2 and T3 (*p* < 0.05). 

### 3.7. Marginal Mucosal Conditions (MMC)

MMC scores are shown in Table 9. A significant reduction was found in both of the groups (*p* < 0.05). Intragroup comparisons showed a significant reduction at T0–T1 for both of the groups (*p* < 0.05). No significant differences were found from intergroup comparisons at each time frame (*p* > 0.05).

## 4. Discussion

In recent years, medical and dental applications of ozone therapy have received particular attention due its beneficial effects, at least in the latter field. Much research has been focused on demonstrating the antimicrobial activity of ozonated products against different kinds of microorganisms [14,15]. Additionally, ozone has been shown to be an effective immunomodulant, anti-hypoxic, anti-inflammatory, and regenerative substance [16].

Considering the applications of ozone therapy for oral diseases, different conditions can be addressed, including difficult-healing wounds, tooth decay, oral lichen planus, gingival inflammation, halitosis, osteonecrosis, pain, endodontic infections, dental hypersensitivity, temporomandibular disorders, and teeth discolorations [16,17].

Implant-prosthetic rehabilitations are nowadays an increasingly frequent solution to addressing total or partial edentulism. The peri-implant gingival tissues are, however, at great risk of undergoing reversible/irreversible inflammation, increasing the risk of failure of the rehabilitation. Based on the latest classification of the 2017 World Workshop [18], the four states of peri-implant tissues are the following: 1, peri-implant health; 2, peri-implant mucositis; 3, periimplantitis; 4, peri-implant soft and hard tissue defects. As regards peri-implant mucositis, it is defined as “an inflammatory lesion of the mucosa surrounding an endosseous implant without loss of supporting peri-implant bone” [1].

The aim of the present report was to evaluate the efficacy of the use of ozonized products compared to chlorhexidine for the in-office/domiciliary management of peri-implant mucositis. The statistically null hypothesis of the present report was partially rejected. Considering PD values, a significant and progressive decrease was found for both the chlorhexidine and the ozonized oil-based gel groups with no significant intergroup differences. A similar trend was also found for SI and for MMC. As regards the other indexes, a significant intragroup decrease was found in both groups and, additionally, significant intergroup differences were also found. Specifically, PI values were significantly lower at three months (T2) for the group following the ozonized oil-based gel protocol (*p* < 0.05). For BoP, the values in the trial group were significantly lower than the control group at every timepoint following the baseline (*p* < 0.05). Finally, BS was significantly lower at three and six months in the trial group (*p* < 0.05). On the basis of these results, the proposed protocol based on the in-office and domiciliary use of the ozonized oil-based gel is effective for the treatment of peri-implant mucositis, with greater efficacy than chlorhexidine when considering specific periodontal indexes.

When considering the research published to date, it is notable that only two experimental studies have been conducted to evaluate the efficacy of the ozonized oil-based gel therapy for the management of peri-implant mucositis, as reported in a recent literature review on adjuvant systems in non-surgical peri-implant treatment [8]. One randomized clinical trial was conducted to compare ozone water and pure water administration in peri-implant mucositis sites [16]. In the study, patients suffering from mucositis [26] were randomly assigned to one of two professional oral hygiene protocols regardless of the pathological sites at baseline, and then reevaluated after 1 and 2 months. The Trial group was submitted to an ozonized water treatment, whereas the Control group underwent a pure water treatment. These interventions were carried out using the same professional irrigator, without differences either in color or in taste between the two substances delivered. At every timepoint, Probing Pocket Depth (PPD), PI, BoP, and BS were recorded. When considering intragroup differences, in the Trial group, ozonized water significantly and progressively decreased all the clinical indexes tested, except for PI in the period T1–T2 (*p* < 0.05); no significant differences occurred for the Control group. In spite of this, no significant intergroup differences were observed between the two treatments. 

The other randomized controlled trial was conducted to evaluate the effect of subgingival applications of ozone and/or hydrogen peroxide on peri-implant mucositis [19]. The subsequent experimental protocols were randomly applied for 60 s to the implant sites on days 0, 7, and 14: (1), O_2_ and 0.9% NaCl (control group), (2) O_2_ and H_2_O_2_ (3%), (3) O_3_ and 0.9% NaCl, and (4) O_3_ and H_2_O_2_ (3%). At days 0, 7, 14, and 21, plaque, gingival, and bleeding indices were recorded. According to the authors, both treatments based on ozone led to an increase in gingival health indexes, and at the same time, both controlled bleeding better than the other tested protocol.

When considering the comparison between ozone and chlorhexidine, a previous study evaluated their respective efficacies for periodontal disease [15]. Ten patients were treated with Scaling and Root Planing (SRP) plus chlorhexidine gel (control sites) and with SRP plus ozone hydrogel (Trial sites). After 1 (T1) and 3 months (T2) from baseline (T0), the following indexes were registered: PPD, clinical attachment level (CAL), Gingival Index (GI), PI, and BoP. Using ozonized hydrogel in addition to SRP did not exhibit significant differences when compared to conventional SRP plus chlorhexidine. Chlorhexidine showed a higher efficacy than ozone in reducing CAL and GI at T2. Despite the fact that these results seem to be in contrast with those in the current report, it is important to restate that different clinical situations were considered (periodontal disease versus peri-implant mucositis). 

Further studies also focused on comparison between ozonized olive oil and chlorhexidine as an adjunct to nonsurgical periodontal therapy for the treatment of periodontitis. In particular, Nambiar et al. selected 30 individuals for a split-mouth randomized controlled trial [20]. Along with SRP, the local application of chlorhexidine and ozonated olive oil was carried out. PPD, relative attachment loss, and Sulcus Bleeding Index (SBI) were measured before and 3 months after the treatment. According to the results of the study, the trial treatment could significantly enhance the outcomes of SRP for the treatment of periodontal diseases. Similar results were also found by other research groups and are also reported in literature reviews [21,22,23,24,25,26,27,28].

Further studies were conducted to compare the efficacy of chlorhexidine and ozone on different conditions. For example, Acikan et al. [29] evaluated the effects of topical application of chlorhexidine, ozone, and metronidazole on palatal wound healing. The authors concluded that the use of all the treatments resulted in enhanced histological wound healing. Moreover, the authors suggested that ozone supplementation could be an alternative therapy to chlorhexidine in impaired wound healing in diabetes mellitus. Kist et al. [30] utilized two disinfection protocols respectively consisting of ozone gas and hypochlorite/chlorhexidine. According to the results obtained, the authors suggested that ozone gas could be regarded as a possible alternative disinfection agent within the root canal treatment of apical periodontitis.

The results of the current study show that both chlorhexidine and the ozonized oil-based gel appear to be effective treatment modalities for peri-implant mucositis, with a better efficacy for the latter on specific clinical indexes (PI, BoP, and BS). As to the limitations of the present study, it should be considered that factors such as the kind of implant, the period since its insertion, and the influence of possible anti-inflammatories among participants were not considered, thus reducing the homogeneity of the two random groups at the starting point. Moreover, Cohen’s kappa coefficient for intra-rater reliability was not assessed. This might hide a measurement bias, especially for PPD, which is generally associated with highly variability between operators assessing this clinical index. 

On the basis of these considerations, the in-office and domiciliary ozonized hydrogel tested in this study seems to be a valid support for the maintenance of implants and the care of tissues surrounding them. Future research should be conducted with a longer follow-up and comparing ozonized oil-based gel with further adjuvant non-surgical approaches such as the use of laser, glycine/erythritol, and probiotics.

## 5. Conclusions

Both chlorhexidine and ozone seem to be valid aids for the in-office and domiciliary treatment of peri-implant mucositis. In particular, the ozonized hydrogel demonstrated a better efficacy for specific clinical peri-implant indexes (PI, BoP, and BS). Further studies should be conducted to evaluate the efficacy of the ozonized hydrogel on the basis of specific factors related to the implant-prosthetic rehabilitation, as well as to compare ozone therapy with other non-surgical adjuvant therapies.

## Figures and Tables

**Table 1:** Inclusion and exclusion criteria.

Inclusion criteria	Age 18–70 years Presence of 2 peri-implant mucositis sites per quadrant with PD > 5 mm; two contralateral quadrants with 2 sites per quadrant were required for the split-mouth study No systemic, metabolic, or autoimmune disease Compliant patients
Exclusion criteria	Implants with peri-implantitis diagnosed by periapical radiographs Patients with periodontitis Neurologic, psychiatric, and mental diseases Patients who took bisphosphonates in the last 12 months Patients taking antibiotics Pregnant and breastfeeding women Patients undergoing anticancer treatment

**Table 2:** Materials tested in the study.

Gel	Manufacturer	Composition
Ozoral Gel and Ozoral Pro	Innovares Srl, Sant’Ilario d’Enza, RE, Italy	Aqua/water, ozonized sunflower seed oil/Heliantus annuus (sunflower) seed oil (product with ozone), aroma/flavor, glycerin, carbomer, polycarbophil, sodium hydroxide, sodium saccharin, illicium verum fruit/seed oil/illicium verum (anise) fruit/seed oil, glyceryl caprylate, tocopherol, ascorbyl palmitate, disodium EDTA, limonene, linalool.
Curasept Periodontal Gel (with 1% chlorhexidine)	Curasept S.p.A, Saronno, VA, Italy	Purified water, propylene glycol, hydroxy ethyl cellulose, PVP/VA copolymer, PEG-40 hydrogenated castor oil, chlorhexidine digluconate, sodium acetate, aroma, acetic acid, sodium metabisulfite, ascorbic acid.

**Table 3:** Demographic data of the study sample at the baseline (T0).

Sex	n (%)	Mean Age (SD)	Min.	Max.
Males	16 (53.33%)	59.14 (7.25)	37	70
Females	14 (46.67%)	58.94 (9.38)	44	69

**Table 4:** Descriptive statistics of Probing Depth (PD) measurements.

Group	Time	Mean	St. Dev.	Min.	Median	Max.	Significance *
Control (Chlorhexidine)	T0	6.55	1.18	5.00	6.00	10.00	A
	T1	5.22	1.17	3.00	5.00	8.00	B
	T2	4.49	1.16	2.00	4.00	7.00	C
	T3	4.23	1.13	2.00	4.00	7.00	D, E
Trial (Ozonized oil-based gel)	T0	6.80	1.21	5.00	7.00	9.00	A
	T1	5.38	1.36	3.00	5.00	9.00	B
	T2	4.41	1.40	2.00	4.00	9.00	C, D
	T3	3.92	1.42	2.00	4.00	8.00	E

* Means with same letters do not show statistically significant differences (*p* < 0.05).

**Table 5:** Descriptive statistics of plaque index (PI).

Group	Time	Mean	St. Dev.	Min.	Median	Max.	Significance *
Control (Chlorhexidine)	T0	84.93	16.74	45.00	89.50	100.00	A
	T1	49.93	18.03	20.00	46.50	100.00	B
	T2	50.13	16.45	15.00	46.50	88.00	B
	T3	47.20	19.43	15.00	42.00	81.00	B, C
Trial (Ozonized oil-based gel)	T0	84.27	16.24	45.00	87.50	100.00	A
	T1	48.63	18.43	20.00	44.00	100.00	B, C
	T2	45.10	18.23	0.00	43.00	79.00	C
	T3	42.03	21.13	0.00	41.00	83.00	C

* Means with same letters do not show statistically significant differences (*p* < 0.05).

**Table 6:** Descriptive statistics of Bleeding on Probing (BoP).

Group	Time	Mean	St. Dev.	Min.	Median	Max.	Significance *
Control (Chlorhexidine)	T0	44.62	26.94	8.33	44.00	100.00	A
	T1	27.07	17.24	0.00	23.68	55.00	B
	T2	18.38	12.32	1.19	19.34	40.25	C
	T3	18.57	15.86	1.10	18.00	83.30	B, C, D
Trial (Ozonized oil-based gel)	T0	46.14	27.09	5.95	42.54	100.00	A
	T1	21.72	13.66	0.00	21.00	48.00	C
	T2	13.31	9.12	0.00	11.95	37.10	D
	T3	9.95	7.17	0.00	10.00	29.40	E

* Means with same letters do not show statistically significant differences (*p* < 0.05).

**Table 7:** Descriptive statistics of Suppuration Index (SI).

Group	Time	Mean	St. Dev.	Min.	Median	Max.	Significance *
Control (Chlorhexidine)	T0	0.16	0.37	0.00	0.00	1.00	A, B
	T1	0.13	0.34	0.00	0.00	1.00	A, B, C
	T2	0.05	0.23	0.00	0.00	1.00	B, C
	T3	0.04	0.20	0.00	0.00	1.00	C
Trial (Ozonized oil-based gel)	T0	0.17	0.38	0.00	0.00	1.00	A
	T1	0.07	0.26	0.00	0.00	1.00	A, C
	T2	0.04	0.19	0.00	0.00	1.00	C
	T3	0.02	0.16	0.00	0.00	1.00	C

* Means with same letters do not show statistically significant differences (*p* < 0.05).

**Table 8:** Descriptive statistics of Bleeding Score (BS).

Group	Time	Mean	St. Dev.	Min.	Median	Max.	Significance *
Control (Chlorhexidine)	T0	2.13	0.66	1.00	2.00	3.00	A
	T1	1.81	0.75	0.00	2.00	3.00	A, B
	T2	1.56	0.76	0.00	2.00	3.00	B, C
	T3	1.31	0.68	0.00	1.00	2.00	C, D
Trial (Ozonized oil-based gel)	T0	2.05	0.71	1.00	2.00	3.00	A
	T1	1.64	0.94	0.00	2.00	7.00	B, C
	T2	1.17	0.70	0.00	1.00	3.00	D, E
	T3	0.85	0.67	0.00	1.00	2.00	E

* Means with same letters do not show statistically significant differences (*p* < 0.05).

**Table 9:** Descriptive statistics of Marginal Mucosal Conditions (MMC).

Group	Time	Mean	St. Dev.	Min.	Median	Max.	Significance *
Control (Chlorhexidine)	T0	2.29	0.49	1.00	2.00	3.00	A
	T1	1.63	0.75	0.00	2.00	3.00	B
	T2	1.32	0.60	0.00	1.00	2.00	B, C, D
	T3	1.09	0.68	0.00	1.00	2.00	D
Trial (Ozonized oil-based gel)	T0	2.27	0.47	1.00	2.00	3.00	A
	T1	1.61	0.71	1.00	1.00	3.00	B, C
	T2	1.22	0.63	0.00	1.00	2.00	C, D
	T3	0.96	0.72	0.00	1.00	2.00	D

* Means with same letters do not show statistically significant differences (*p* < 0.05).

## Data Availability

Data are available upon request to the corresponding authors.

## Abbreviations

PD	Probing Depth
PI	Plaque Index
SI	Suppuration Index
BS	Bleeding Score
MMC	Marginal Mucosa Condition
BoP	Bleeding on Probing
SRP	Scaling and Root Planing
